# Factors determining poor prognostic outcomes following diabetic hand infections

**DOI:** 10.12669/pjms.313.6858

**Published:** 2015

**Authors:** Bilsev Ince, Mehmet Dadaci, Abdullah Arslan, Zeynep Altuntas, Mustafa Kursat Evrenos, Mehmet Fatih Karsli

**Affiliations:** 1 Bilsev Ince, MD. Department of Plastic & Reconstructive and Aesthetic Surgery, Faculty of Meram Medicine, Necmettin Erbakan University, 42080 Meram, Konya, Turkey; 2 Mehmet Dadaci, MD. Department of Plastic & Reconstructive and Aesthetic Surgery, Faculty of Meram Medicine, Necmettin Erbakan University, 42080 Meram, Konya, Turkey; 3 Abdullah Arslan, MD. Department of Underwater and Hyperbaric Medicine, Faculty of Meram Medicine, Necmettin Erbakan University, 42080 Meram, Konya, Turkey; 4 Zeynep Altuntas, MD. Department of Plastic & Reconstructive and Aesthetic Surgery, Faculty of Meram Medicine, Necmettin Erbakan University, 42080 Meram, Konya, Turkey; 5 Mustafa Kursat Evrenos, MD. Department of Plastic & Reconstructive and Aesthetic Surgery, Faculty of Medicine, Celal Bayar University, Manisa, Turkey; 6 Mehmet Fatih Karsli, MD. Dr. Sami Ulus Maternity-Children’s Health and Diseases Training and Research Hospital, Ankara, Turkey

**Keywords:** Diabetic hand, Hand infection, Amputation, Peripheral neuropathy, HbA1c, end-stage renal disease

## Abstract

**Background and Objective:**

Hand ulcers are seen in a small percentage of patients with diabetes. The predisposing factors of diabetic hand varies between different countries. However, the effects of predisposing factors on prognosis are not clear in diabetic hand infections. In this study, our aim was to determine the effects of predisposing factors on poor prognostic outcomes in patients with diabetes mellitus.

**Methods:**

Thirty-four patients with diabetes mellitus who were treated and followed up for a hand infection in between 2008 and 2014 were investigated retrospectively. Patients were evaluated according to predisposing factors defined in the literature that included disease period, age, gender, admission time, presence of neuropathy, smoking habits, HbA1c levels at admission time, peripheral vascular disease, end-stage renal disease (ESRD), and trauma. Death and minor/major amputation cases during treatment were defined as poor prognosis.

**Results:**

Patients who had ESRD, peripheral neuropathy, or an HbA1c level greater than 10% had significantly higher amputation rates.

**Conclusions:**

Peripheral neuropathy, ESRD, and HbA1c levels greater than 10% at the time of admission were determined as poor prognosis criteria for diabetic hand treatment.

## INTRODUCTION

Diabetes mellitus is a multisystemic disease that affects multiple organs. Hyperglycemia associated with diabetes represses cellular immunity, causes microangiopathy, and prevents chemotaxis and phagocytosis, so infection and poor wound healing occurs.^1^-^4^ Peripheral neuropathy and circulatory disorders facilitate wound formation and make the healing process difficult.^5^

Foot ulcers are seen in 9.7% of patients with diabetes mellitus, while hand ulcers are seen in a small percentage (0.37%) of patients.^6^ Hand ulcers of patients with diabetes mellitus can be infected and turn into localized cellulitis which may lead to severe hand sepsis, or even become gangrenous.^7^,^8^ Because the functions of the hands are crucial, improving a diabetic hand is an important issue and prognostic factors should be identified.^9^,^10^

The publications on this subject are limited, the predisposing factors of diabetic hand varies between different countries. End-stage renal disease (ESRD), foot wounds, lower extremity neuropathy, and smoking are associated with diabetic hand infections in Western countries.^11^,^12^ In China, diabetic hand infections are associated with poor blood glucose regulation, long-term diabetes, old age, and late admission to the hospital.^6^ While in Africa, they are associated with poor blood glucose regulation, late admission to the hospital, gender, low socioeconomic status, hand trauma, and insect bites.^7^ However, the effects of these predisposing factors on prognosis are not clear in diabetic hand infections.

In this study, we evaluated the treatment results of cases diagnosed as diabetic hand infections. Our aim was to determine the effects of predisposing factors on poor prognostic outcomes in patients with diabetes mellitus.

## METHODS

Thirty-four patients with diabetes mellitus who were treated and followed up for a hand infection between 2008 and 2014 were investigated retrospectively from our clinic’s patient file archive. Patients living in the same geographic region with diabetic hand infections were included to the study. We excluded the patients with amputations, who had open wounds due to burn wound infection. The socio-economic status of the patients, smoking story and the comorbities were examined. This study did not require institutional review board (IRB) approval because this therapy is a standard treatment for hand infections in our institution.

Routine biochemical analysis along with evaluation of HbA1c levels and total blood count were performed in all patients immediately after their admission. Infection markers and blood glucose levels were monitored regularly in all patients. All patients were treated with insulin to regulate the blood glucose levels independent of their previous treatments, and the blood glucose levels were monitored four times daily. According to the results of blood glucose monitoring, patients were referred to endocrinology clinic.

Hand lesions were classified as felons, deep palmar space infections, septic arthritis, cellulitis, and osteomyelitis. Osteomyelitis is diagnosed according to the direct radiography images. Diagnosis is confirmed with magnetic resonance imaging (MRI) scan in doubtful cases.

Patients who did not have cellulitis were operated on under local anesthesia. Surgical debridement was performed and/or abscesses were drained. Deep tissue cultures were obtained during the surgery and prophylactic wide-spectrum antibiotics effective against gram-negative anaerobes were administered intravenously. Antibiotics were changed according to the results of cultures, if necessary. Patients were followed according to the gross appearance of the wounds, wound cultures and infection markers. Antibiotic doses were adjusted according to the creatinine levels of the patients. The wounds were dressed twice daily. Serial debridements were performed when necessary during the follow-up period. Despite all surgical and medical treatments, finger and/or hand amputations were performed in some patients because of progressive infection and circulation problems. Amputations distal to the metacarpal joints were defined as minor amputation while, amputations proximal to the metacarpal joints were defined as major amputation. Patients with end-stage renal disease (ESRD) received hemodialysis three times weekly.

Patients were evaluated for retinopathy, nephropathy, and neuropathy and were referred to the appropriate department if these disorders were detected. Patients were evaluated according to the predisposing factors defined in the literature that included disease period, age, gender, socio-economic status, admission time to hospital, presence of neuropathy, smoking habits, HbA1c levels at admission time, peripheral vascular disease, end-stage renal disease, and trauma. Hand functions were checked after treatment. Death and minor/major amputation cases during treatment were defined as poor outcome.

### Statistical Analysis

The statistical significance of the differences between mean values was analyzed using SPSS 18.0 (USA) statistical software. The relationship between disease period, age, gender, admission time to hospital, presence of neuropathy, smoking habits, HbA1c levels at admission time, peripheral vascular disease, end-stage renal disease, trauma, and amputation was analyzed using the Mann-Whitney U test. The relationship between education level, income level and amputation was analyzed using the Chi-square test. A p value of ≤0.05 was considered to be statistically significant.

### Ethical Review Committee Statement

This study conformed to the Helsinki Declaration.

## RESULTS

Thirty-four patients (23 male, 11 female) were followed for an average of 25 months (range, 6-46 months). The mean of their ages was 61 years (range, 33–78 years) (Table-I). The average duration between onset of symptoms and hospital admission was 9 days (range, 2–16 days), and the average duration of hospitalization was 12.2 days (range, 5–23 days). The average HbA1c level at the time of admission was 10.1 (range, 7–13), and the average duration of diabetes was 8.2 years (range, 0–14 years). Fourteen patients were non-smokers and eighteen patients had smoking history. Neuropathy of the feet was present in thirteen patients. End-stage renal disease related to diabetic nephropathy was seen in eight patients. Nephropathy and retinopathy were present in twenty-four patients. The functional loss of the hands was 23.5% (34/8) (6 amputation, 2 ankylosis). The educational level of the patients was; seven patients graduated from high school or university, eleven patients graduated from primary school to high school, and sixteen patients graduated from primary school (Table-II).

**Table-I:** Datas of patients.

Patient	Sex-age	Application time (day)	Neuropathy	Smoking	Diabetes time	HBa1c	Hospitalization (day)	Result
1	M-65	8	+	+	11	13	20	Minor Amputation
2	M-66	16	-	-	9	8.7	12	Graft
3	M-33	7	-	-	12	10.4	13	Recovery
4	M-64	11	-	+	7	9	12	Graft
5	F-62	4	-	+	9	12.9	15	Recovery
6	M-53	9	+	-	11	13.1	16	Major amputation
7	M-38	8	-	+	12	11.7	13	Recovery
8	F-67	16	+	-	14	10.6	17	Minor Amputation
9	F-66	13	-	+	8	8.9	7	Curettage
10	M-78	10	+	-	15	11.2	17	Curettage
11	M-66	6	+	-	13	12.3	23	Minor Amputation
12	F-63	2	-	+	10	9.1	15	Recovery
13	M-51	12	-(trauma)	+	0	11.8	11	Flap
14	M-57	4	+(trauma)	-	0	8.6	6	Flap
15	M-67	6	-	-	13	9.3	8	Recovery
16	F-62	4	-	+	4	7.7	5	Recovery
17	M-56	12	-(trauma)	+	5	8.1	12	Flap (Funcional loss)
18	F-72	9	+	-	8	12.1	11	Minor Amputation
19	F-58	13	-	+	7	8.3	10	Flap
20	M-69	6	-	+	11	9.2	7	Curettage
21	M-49	10	-	+	0	11.1	13	Graft
22	M-74	8	+	-	12	8.2	10	Recovery
23	M-46	7	-	+	0	8.4	7	Curettage
24	M-63	6	+	-	5	11.8	9	Graft
25	F-62	5	+	-	6	9.9	11	Graft
26	M-59	7	-	+	4	11.7	17	Minor Amputation
27	M-71	2	+	+	9	9.2	13	Graft
28	F-47	5	+	-	13	9.8	12	Graft
29	M-66	12	+	-	11	10.1	10	Recovery
30	M- 65	7	-	+	6	7.9	10	Graft
31	F-57	8	-	+	7	11	17	Recovery
32	M-58	10	-	-	9	8.8	14	Recovery
33	F- 55	6	-	+	12	9.5	5	Recovery
34	M-65	11	-	-	3	9.7	16	Curettage (Funcional loss)

**Table-II:** Education and income levels of patients.

Patient education level	Income level	The No. of patients	The No. of amputation	The No. of functional loss
Graduated from primary school	Low	7	2	-
	Medium	8	1	1
	High	1	1	-
Primary-High school	Low	3	-	-
	Medium	6	1	-
	High	2	-	-
Graduated from high school or university	Low	-	-	-
	Medium	3	-	1
	High	4	-	-

Monthly income level: Low ≤ 350 €, medium ≤ 1000 €, high > 1000 €

Polymicrobial infection was observed in fourteen patients. *S. aureus* and *Klebsiella* were the most frequently encountered bacteria in our study. No bacteria were isolated only in one patient.

### Type of hand lesions

### Osteomyelitis

**Fig.1:**
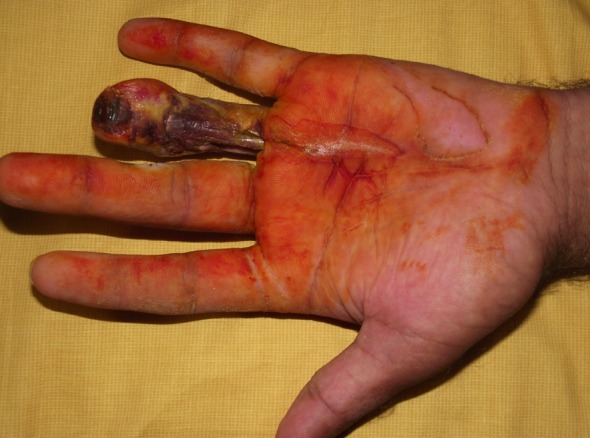
Appearance of necrosis on the ring finger.

Five of the patients with osteomyelitis on the distal phalanx had curettage, and three of them underwent amputation at the level of the proximal phalanx (Fig.1). All the metacarpal bones and the hand were amputated at the level of the wrist in one patient with osteomyelitis in the distal part of the radius and ulna.

### Felons

Abscesses were drained in 5 patients. One of them was debrided, covered with a reverse-flow flap from the dorsal part of the finger and covered with full-thickness skin graft.

### Trauma

**Fig.2:**
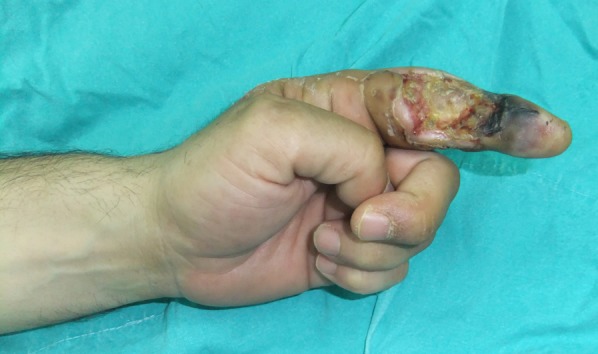
Appearance of necrosis.

Three cases were debrided (Fig.2), covered with a reverse-flow flap from the dorsal part of the hand and covered with full-thickness skin graft.

### Septic arthritis

The second finger was amputated at the level of PIP joint in one patient. The third finger of the left hand was amputated at the middle of the central phalanx in another patient.

### Cellulitis

**Fig.3:**
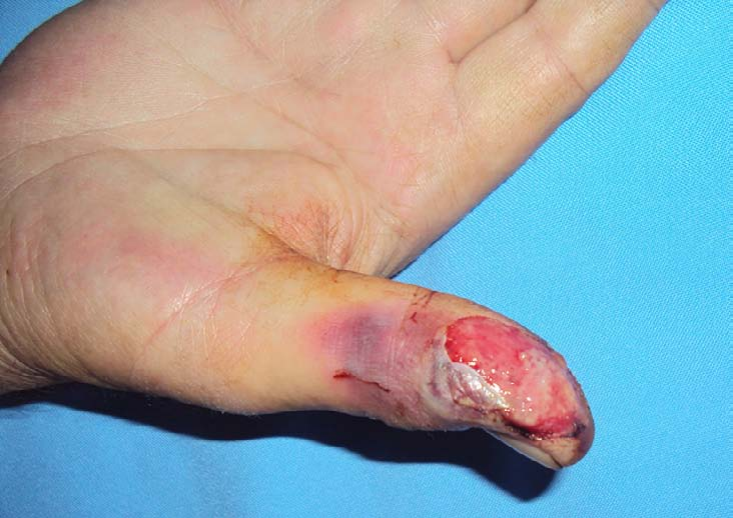
Appearance of open wound on the thumb.

Five patients had cellulitis or open wound (Fig.3). Antibiotic treatment was given to these patients. Full thickness skin grafts were applied after debridement in three patients.

### Deep palmar space infections

**Fig.4:**
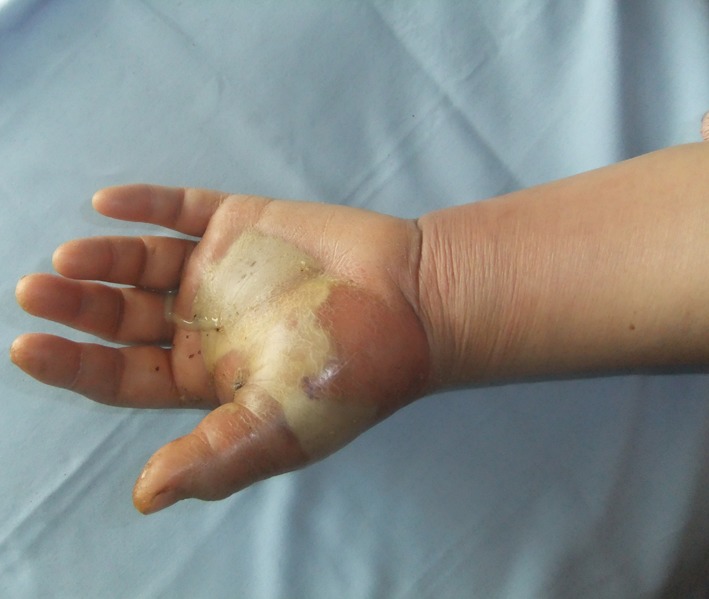
Appearance of deep palmar space infection.

Abscesses were drained in 10 patients (Fig.4). Full thickness skin grafts were applied after debridement in 5 patients.

### Outcomes

None of the patients in this study died. Minor amputation was performed in five patients and major amputation was performed in one. Hospitalization time was significantly longer in patients who were admitted to hospital seven or more days after the onset of symptoms; had diabetes mellitus for more than 10 years; had neuropathy, end-stage renal disease, or an HbA1c level greater than 10%; or were 65 years or older (p ≤ 0.05). Patients who had end-stage renal disease, peripheral neuropathy, or an HbA1c level greater than 10% had significantly higher amputation rates (p ≤ 0.05). Neuropathy was significantly more common in individuals who had diabetes for more than 10 years (p ≤ 0.05). Trauma, diabetes duration, gender, smoking habits and peripheral vascular disease were not associated with the rate of sequelae, end-stage renal disease, or hospitalization time (p > 0.05). No statistical difference was found between comorbidies and amputation (p > 0.05). Diabetic hand was seen more in people with low education (p ≤ 0.05). The rate of amputation was high (25%) in people with low educational levels (4/16). However, there was no statistically significant difference between income levels and the incidence of diabetic hand and the amputation rate (p > 0.05). Functional loss was not associated with the duration of diabetes, end-stage renal disease, or admission time to the hospital (p > 0.05).

## DISCUSSION

In our study, we observed that end-stage renal disease, peripheral neuropathy, and an HbA1c level greater than 10% were significantly associated with poor prognosis in diabetic hand infections. Hospitalization time was longer in patients who were admitted to hospital seven or more days after the onset of symptoms; had diabetes mellitus for more than 10 years; neuropathy, ESRD, or HbA1c level greater than 10%; or were 65 years old or more. Diabetic hand cases are rare in the literature compared to foot lesions,^7^ although hand infections are often seen at a younger age.^11^ Usually, admission of patients with a hand infection to the hospital after the onset of the symptoms is delayed and trauma is present in 16% of cases.^11^ The trauma may be minor or caused by animal bites.^12^

Tropical diabetic hand syndrome (TDHS) is one of the diabetic hand infections which can recover without sequelae, but it may also result in loss of hand function, amputation, or death.^7^-^10^,^13^,^14^ Risk factors of TDHS include poor blood glucose regulation, neuropathy, insulin therapy, and malnutrition.^7^ Even though diabetic hand was defined in tropical regions, it may also be seen in western countries.^11^,^12^ Our data indicated that diabetic hand infections were related to education status, awareness of the infection, and blood glucose control rather than climate or geography. The low level of patient education delayed noticing of the infection in patients’ hands, and therefore, the initiation of treatment in diabetic patients.

Hand sepsis is a serious complication of diabetes, but early diagnosis and treatment may lead to adequate recovery.^15^ Prognosis improves when appropriate blood glucose and insulin control, antimicrobial therapy, drainage, and debridement are performed promptly after diagnosis.^6^,^16^ In our study, none of patients with sepsis was observed. The rate of major amputations related to diabetic hand infections is between 0%–13% in the literature,^6^,^7^,^17^ and was 2.9% in our study. Reduced hand function has been detected in 46% of the patients in a previous study.^17^ However, 23.5% of our patients exhibited reduced hand function which may be related to early diagnosis, aggressive and repeated debridement, strict blood glucose regulation, prophylactic wide spectrum antibiotic therapy and prompt change of antibiotic therapy according to culture results, and physiotherapy in our subjects. These can lower frequency of reduced hand function.^6^,^7^,^15^ Although the incidence of death was 0%–100% in previous studies,^6^,^7^,^13^,^17^ all of our patients lived during our follow-up period.

*S. aureus* and *Klebsiella* are the most frequently found bacteria in diabetic hands, but infections are generally polymicrobial^3^,^16^,^18^,^19^ and our results are in accordance with this information.

The most frequent comorbidity of diabetic hand is hypertension.^14^ In addition to the hypertension, CRF, congestive heart failure, and peripheral circulatory disorders were common comorbidities in our patients. These comorbidities was found in our patients with amputations. However, we did not find a significant relationship between the presence of comorbidy and amputation.

A longer duration of diabetes increases the incidence of complications. However, blood glucose monitoring is as important as duration of diabetes for the incidence of complications. In our study diabetes duration was not associated with the rate of sequelae, ESRD, or hospitalization time. Some patients may have had undiagnosed diabetes or were diagnosed long after the disease had actually developed, which may have influenced our observed results.

One of the complications of the diabetes is circulation problem. Circulatory disorders delay wound healing. Although no statistical difference was found between smoking, which is one of the factors known to disrupt circulation, and amputation, amputation rate was found to be increased in patients with HbA1c levels ≥10% in our study. Poorly controlled blood glucose is associated with low socioeconomic status and illiteracy, which also delays the time of hospital admission. Long-term hyperglycemia represses cellular immunity and leaves the body vulnerable to infections.^3^,^4^ Circulation disorder is an important factor delaying wound healing but we think that the primary cause of rapid progress and emergence of infection is the suppression of immunity by hyperglycemia. In our study, insulin was administered to all patients for regulation of blood glucose levels regardless of their prior therapy.

Amputation was significantly more common in individuals with ESRD. Patients who receive dialysis because of ESRD often get arterial calcification and arteriovenous shunts, which may cause circulation problems in the hands and lead to amputation of the upper extremities because of gangrene.^20^

In our study, all patients who underwent a minor amputation had neuropathy, and the patient who had major amputation had peripheral vascular disease in addition to neuropathy. However, we did not find a significant relationship between the presence of neuropathy and major amputation. The reason may be the small number of patients in our study. Presence of peripheral neuropathy, which is a common late-stage complication of diabetes, indicates that diabetes is not adequately controlled, and was defined as a criterion for poor prognosis in our series.

Peripheral vascular disease, peripheral neuropathy, ischemia, diabetic foot infections, and ulcers are well-known risk factors. Abbas et al.^7^ detected 10 patients with peripheral neuropathy and one patient with peripheral vascular disease in their 72-patient series. However, another study claimed that peripheral neuropathy has an important role in the pathogenesis of hand ulcers.^6^ Microangiopathy in diabetes mellitus is related to basal thickening of the small-vessel membrane. It may disrupt the hyperemic response by preventing leukocyte migration after injury,^5^ which increases local ischemia damage and susceptibility to infection.

Although ESRD,^11^,^12^ foot wounds,^11^,^12^ lower extremity neuropathy,^11^,^12^ smoking history,^11^,^12^ poor blood glucose regulation,^6^,^7^ long-term diabetes,^6^ old age,^6^ hand trauma,^7^ insect bites,^7^ female gender,^7^ and late admission to the hospital^6^,^7^ are defined among the predisposing factors, only peripheral neuropathy, ESRD, and HbA1c level greater than 10% at the time of admission were determined as poor prognostic criteria for diabetic hand treatment. Early diagnosis, strict blood glucose regulation, suitable antibiotic therapy, and aggressive-repeated debridement may decrease the incidence of complications in diabetic hand treatment.
